# The Bacterial Oral Microbiome in Children with Congenital Heart Disease: An Extensive Review

**DOI:** 10.3390/pathogens12101269

**Published:** 2023-10-21

**Authors:** Maria Hofmann, Nelly Schulz-Weidner, Norbert Krämer, Torsten Hain

**Affiliations:** 1 Dental Clinic—Department of Paediatric Dentistry, Justus Liebig University, Schlangenzahl 14, 35392 Giessen, Germany; nelly.schulz-weidner@dentist.med.uni-giessen.de (N.S.-W.); norbert.kraemer@dentist.med.uni-giessen.de (N.K.); 2 Institute of Medical Microbiology, Justus Liebig University, Schubertstrasse 81, 35392 Giessen, Germany; torsten.hain@mikrobio.med.uni-giessen.de; 3 German Center for Infection Research (DZIF), Partner Site Giessen-Marburg-Langen, Schubertstrasse 81, 35392 Giessen, Germany

**Keywords:** oral microbiome, children, congenital heart disease

## Abstract

Children with congenital heart disease have poorer oral health compared with healthy children. Oral diseases, such as dental caries and gingivitis, are associated with the oral microbiome. The objective of this review was to find evidence of differences in the bacterial colonization of the oral cavity of children with congenital heart disease (CHD) versus healthy children. A literature review was conducted according to predetermined criteria, including the need for controlled clinical trials. Half of the 14 studies that met the inclusion criteria reported significant differences in bacterial colonization in children with congenital heart disease. A variety of influencing factors were discussed. There is some evidence for alterations in the oral microflora as a result of physiopathological and treatment-related factors in children with CHD, but additional research is required to validate these findings.

## 1. Introduction

Annually, 1.35 million infants are born with congenital heart disease (CHD) [1]. Children with CHD endure invasive surgical procedures, physical limitations, and ambiguous prognoses [2]. Moreover, CHD imposes a financial and emotional burden on affected families [3].

There are several studies that observed that children with CHD have poorer oral health, including more caries [4,5] and gingivitis [4], higher DMFT/DMFS values (higher number of teeth with caries history), a higher number of untreated teeth with dental caries [6,7,8,9], and higher plaque and gingival indices [10,11] compared with healthy control individuals of the same age.

Possible explanations for these associations between a congenital defect, such as CHD, and an acquired disease, such as caries, are that the focus of the affected families is often not primarily on oral health, the parents’ lack of knowledge about adequate oral preventive measures or the disease burden of CHD [5,12].

In order to investigate whether other host factors, such as the individual bacterial colonization of the oral cavity, could be responsible for a higher caries risk in children with CHD, it is necessary to look at the factors of origin of dental caries.

It is known that dental caries are a multifactorial disease. Examples of causal factors include the amount and frequency of dietary intake (particularly of sugars), the individual qualitative and quantitative properties of saliva, oral hygiene practices, the local use of fluorides, and oral health awareness [13].

With respect to oral hygiene practices, tooth brushing [12,14] and the use of fluoridated toothpaste [14] occur less frequently in children with CHD than in healthy children of the same age, whereas the consumption of cariogenic foods [12] is substantially higher in children with CHD. Moreover, parents of children with CHD frequently have a very limited understanding of the significance of oral health [15].

Oral bacteria play a significant role as a cause of oral diseases such as dental caries and inflammatory diseases of the periodontium [13,16].

Concerning dental caries, the bacterial balance in the oral cavity is dominated by acid-tolerant and acid-producing microorganisms, such as *Streptococcus mutans* and *Lactobacillus* spp. (species), which produce a low pH environment that can result in the demineralization of dental tissues [17,18].

*Lactobacillus* spp. are responsible for the progression of carious lesions [19], whereas *Streptococcus mutans* is one of the major causative agents of dental caries [20]. *Streptococcus mutans*, a natural resident of the human oral cavity, can induce changes in the plaque microbiome by synthesizing large amounts of extracellular glucan polymers and acids.

Dominant Lactobacillus spp. in carious lesions are, for example, *Lactobacillus fermentum*, *Lactobacillus rhamnosus*, *Lactobacillus pontis*, *Lactobacillus gasseri*, *Lactobacillus casei*, *Lactobacillus paracasei*, *Lactobacillus salivarius*, *Lactobacillus plantarum*, *Lactobacillus oris* and *Lactobacillus vaginalis* [19,21]. *Lactobacillus acidophilus*, another representative of *Lactobacillus* spp., is a cariogenic bacterium [22] that has a sugar metabolism system functionally similar to that of *Streptococcus mutans* [23].

In gingivitis, a periodontal disease caused by plaque accumulation, the oral microflora in the biofilm favors *Actinomyces* spp. and capnophilic and obligate anaerobic bacteria such as *Fusobacterium* spp., *Capnocytophaga* spp., and *Prevotella* spp. [17].

A congenital heart defect can be a risk factor for the development of a brain abscess due to the dissemination of odontogenic bacteria into the bloodstream during dental procedures [24]. In addition, many patients with CHD are susceptible to infective endocarditis (IE) due to oral infections [25] and dental procedures [26]. IE, a disease of the heart tissue that can be predisposed because of CHD [27], shows a high mortality rate [28] and is one of the most severe complications caused by oral bacteria-induced bacteremia [27].

HACEK (*Haemophilus* spp., *Aggregatibacter actinomycetemcomitans*, *Cardiobacterium hominis*, *Eikenella corrodens,* and *Kingella kingae*) organisms, which are Gram-negative bacteria that are part of the normal oral microflora [29,30], are responsible for 1% to 2% of cases of IE [29]. IE caused by HACEK organisms mainly impacts patients with pre-existing heart disease or prosthetic heart valves [31].

In order to prevent complications such as IE, antibiotic prophylaxis is recommended for patients at high risk, such as those with specific cardiac conditions [26,32]. This could lead to the hypothesis that changes in the bacterial spectrum of the host organism can occur because of the frequent intake of antibiotics in children with cardiac conditions.

In addition, it is assumed that physiopathological processes associated with CHD may also result in alterations in bacterial colonisations within the human body. For children with CHD and heart failure, alterations in the intestinal bacterial spectrum due to heart failure have already been demonstrated [33].

For newborns with critical CHD, Huang et al. (2022) have already demonstrated a correlation between this congenital disease and an altered gut microbiome. According to the authors, this altered gut microbiome is associated with an imbalance of the immune system and unfavorable clinical outcomes [34]. Since the oral cavity serves as the entry point of the intestinal tract, the question arises whether the oral microbiome is also determined by the congenital general disease and whether this predisposition is a potential cause for poorer oral health in children with CHD. So far, there are no published data on this.

This article’s primary objective is to explore whether there is evidence of differences in the bacterial colonization of the oral cavity between children with CHD and healthy children.

## 2. Materials and Methods

A literature review was carried out until August 2023 using the database “PubMed” (https://pubmed.ncbi.nlm.nih.gov/; last accessed on 1 August 2023). The research question “Is there any evidence of differences in the bacterial colonization of the oral cavity between children with CHD and healthy children?” was generated using the “PICO” scheme (Table 1).

**Figure 1:**
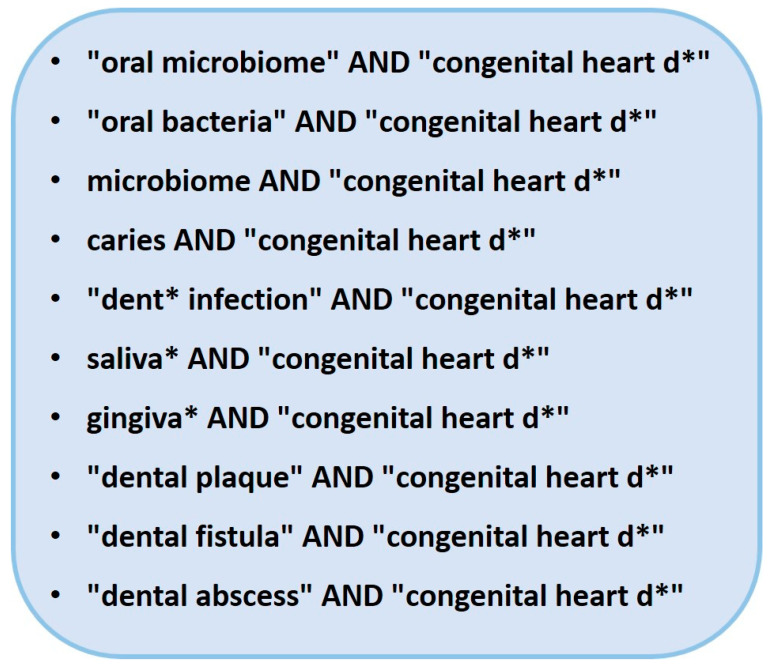
Search terms for the literature search in PubMed (https://pubmed.ncbi.nlm.nih.gov/; last accessed on 1 August 2023).

Since the literature search with MeSH (Medical Subject Headings) terms analogous to the research question according to the PICO scheme did not yield any results, the search terms shown in Figure 1 were used. These search terms should represent all studies that examine children with congenital heart disease or congenital heart defects for bacterial colonization of the oral cavity. Furthermore, search items were added that referred to various inflammatory processes of the oral cavity and/or to the sample medium.

There were no limitations on the language or date of publication in the literature. Included were clinical trials that studied the oral bacteria of children with CHD. In addition, an active control population without any heart defects had to be part of the trials. Only studies reporting the procedure of sampling and detection of the examined bacteria were included.

Case reports, retrospective analyses, non-human trials, or studies with test subjects older than eighteen years of age were excluded.

The screening of the literature was carried out by one examiner and was repeated three times.

**Figure 2:**
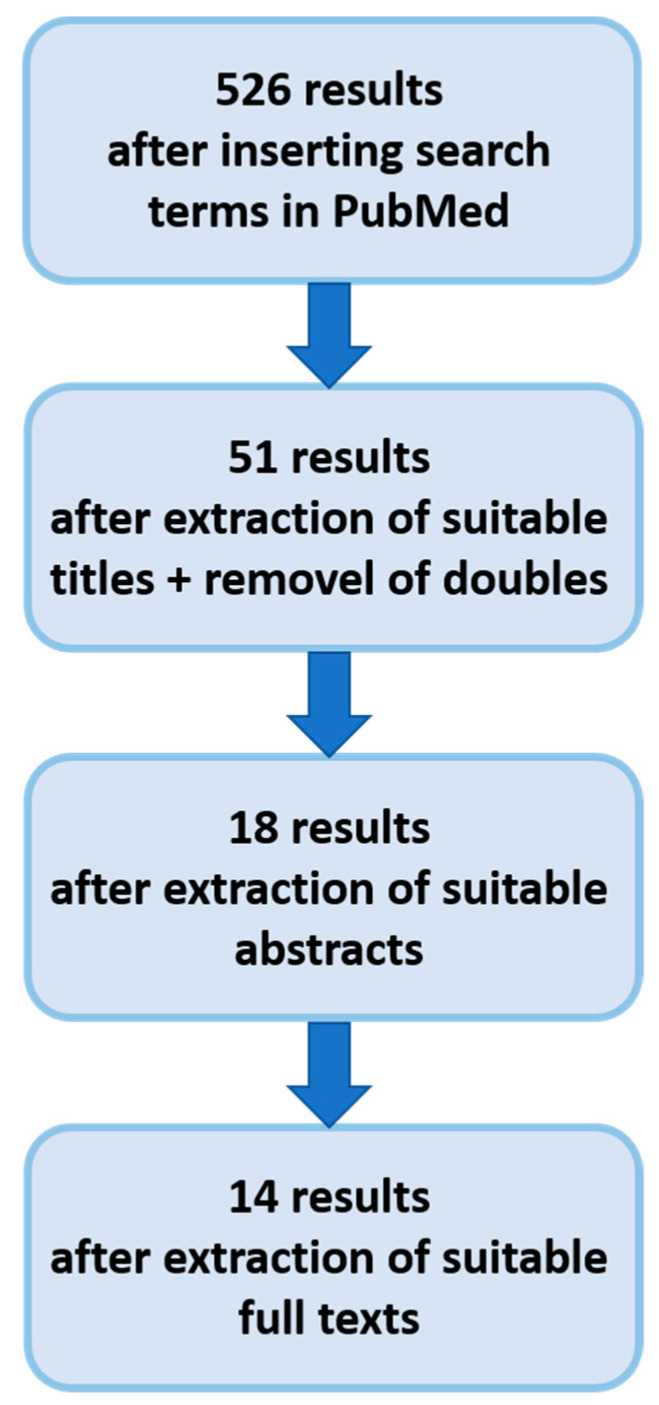
Taxonomy of the literature search.

The taxonomy of the literature research process is depicted in Figure 2.

## 3. Results

For this review article, fourteen studies spanning the years 1986 to 2021 were included.

Seven of these studies come from industrialized nations, such as the United States of America [35,36], the United Kingdom [6,37], Sweden [38,39] or Germany [40]. Three of the included studies were conducted in emerging countries, such as Turkey [41,42] or Brazil [43]. Four of the studies presented are from developing countries, such as Sudan [22], Iran [44,45] or India [11].

Eleven of the fourteen included studies [6,11,35,36,37,38,39,42,43,44,45] examined bacteria using bacterial culture on various specific agar plates in conjunction with the determination of the number of colony-forming units (CFUs). One of these eleven investigations described the use of additional Gram-staining to distinguish between Gram-positive and Gram-negative bacteria [37]. A second study used species-specific 16S rRNA gene sequences for the identification of specific bacteria [42]. A third study reported on a DNA probe test that followed the bacterial culture [36].

The remaining three studies [22,40,41] identified bacteria using DNA extraction, amplification, and sequencing.

In nine studies, saliva was used to detect oral bacteria [6,38,39,41,42,43,44,45], dental supragingival plaque in three [6,22,37], subgingival samples from the gingival sulcus in two [35,36], and carious dentinal material in one [40] study. Another study examined cardiac tissue samples from the CHD group for the presence of particular bacteria [41].

**Figure 3:**
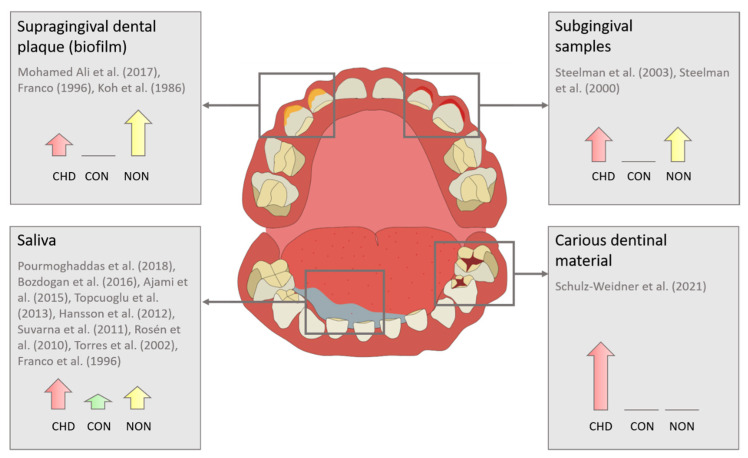
Investigated media and distribution of differences in bacterial colonization in relation to the number of available studies (CHD [red arrow]: congenital heart disease, CON [green arrow]: control, NON [yellow arrow]: none of the groups). Sources: Ajami et al. (2015) [44], Bozdogan et al. (2016) [41], Franco (1996) [6], Hansson et al. (2012) [38], Koh et al. (1986) [37], Mohamed Ali et al. (2017) [22], Pourmoghaddas et al. (2018) [45], Rosén et al. (2010) [39], Schulz-Weidner et al. (2021) [40], Steelman et al. (2000) [35], Steelman et al. (2003) [36], Suvarna et al. (2011) [11], Topcuoglu et al. (2013) [42], Torres et al. [43]. © Figure 3 by Dr. Maria Hofmann.

A graphical representation of the media tested for bacteria and the differences in bacterial colonization between groups in relation to the number of studies found is given in Figure 3.

Eleven of the studies [6,11,22,35,37,38,39,42,43,44,45] focused on oral *Streptococcus* spp., with the majority focusing on *Streptococcus mutans* [6,22,35,38,39,42,43,44,45]. Seven of these eleven studies also looked for *Lactobacillus* spp. [6,22,38,39,42,43,45], one for amoxicillin-resistant *Streptococcus* spp. [37], and one for the total viable number of bacterial CFUs [39]. Three articles [35,36,41] reported the identification of microorganisms belonging to the HACEK bacterial group.

Only one study [40] did not focus solely on the identification of predefined bacterial species but instead examined the most comprehensive coverage of the microbiome possible.

Seven studies reported statistically significant differences in bacterial colonization between groups of children with CHD and groups of healthy children [22,38,39,40,43,44,45], three studies did not find any statistically significant differences [6,41,42], and four studies mention differences but do not report statistical significance for every variable investigated [11,35,36,37].

The characteristics of the studies are summarised in Table 2.

## 4. Discussion

### 4.1. Distribution of Oral Bacteria in Children with CHD

The findings of this review suggest that the oral bacterial spectrum is altered in children with CHD compared with healthy controls. Suvarna et al. (2011), for instance, discovered a higher number of *Streptococcus mutans* CFUs in the saliva of infants with CHD compared with healthy controls [11].

Hansson et al. (2012) were also able to demonstrate substantially higher *Streptococcus mutans* levels in children with CHD than in healthy controls at twelve months of age but not at six or nine months [38]. The authors propose that factors such as increased frequency of meals, changes in salivary composition due to the use of diuretics, and increased use of antibiotics could explain the significant differences at 12 months of age [38]. Regarding the fact that the first teeth erupt between six and twelve months of age [46] and that the awareness of the importance of good oral hygiene among parents of children with CHD is very low [15], another assumption could be the neglect of dental hygiene, especially considering that the majority of children with CHD in the study by Hansson et al. (2012) have already undergone corrective surgery within the first twelve months of life [38] and that this risky intervention, with all its implication for the child’s potential health.

Franco et al. (1996), in contrast, found no significant difference in the distribution of the number of CFUs of plaque or saliva *Streptococcus mutans* or *Lactobacillus* spp. between children with cardiovascular disease and healthy controls [6]. As a possible explanation, the authors noted that the control group was comprised of individuals with more severe dental disease than the general population. As previously mentioned, children with CHD frequently have poorer oral health than healthy children [4,5,7,8,9,10,11].

### 4.2. The Microbiome of Dental Caries and Its Implications in Children with CHD

Schulz-Weidner et al. (2021) [40] answered the question of whether the microbial composition of dental caries itself differs in this population with higher oral healthcare requirements. The authors were able to demonstrate statistically significant differences in the bacterial composition of carious dentinal samples between preschool children with CHD and healthy individuals [40]. *Fusobacterium* spp., *Prevotella* spp., *Capnocytophaga* spp., and *Oribacterium* spp. were found to be substantially more abundant in the CHD group [40] at the level of operational taxonomic units (OTUs).

As previously described, *Fusobacterium* spp., *Capnocytophaga* spp., and *Prevotella* spp. are associated with gingivitis [17], so the findings of Schulz-Weidner et al. (2021) may account for the higher incidence of gingivitis in children with CHD [4].

This hypothesis is supported by the findings of Mohamed Ali et al. (2017), who observed a positive correlation between the gingival index and *Tanerella forsythia*, *Camphylobacter rectus*, and various *Fusobacterium* spp. in only children with CHD. In the study by Mohamed Ali et al. [22], children with CHD also had higher caries and gingivitis incidences.

In addition, Mohamed Ali et al. (2017) discovered a positive correlation between the amount of CFUs of *Streptococcus mutans* and the number of decayed teeth in both children with CHD and healthy controls [22]. Pourmoghaddas et al. (2018) were unable to demonstrate a significant correlation between a higher amount of CFUs of bacteria (*Streptococcus mutans* or *Lactobacillus* spp.) and other factors, such as the number of decayed, missing, or filled teeth as a result of caries, or the gingival bleeding index [45]. In this context, it is important to note that in the study by Pourmoghaddas et al. (2018), the healthy control group had a substantially higher carbohydrate intake than the examined children with CHD [45].

Mohamed Ali et al. (2017) [22] were the only authors reporting on the examination of one specific *Lactobacillus bacterium*: *Lactobacillus acidophilus*. *L. acidophilus* is a bacterium that is commonly found in saliva samples of subjects with dental caries [21]. The fact that Mohamed Ali et al. (2017) examined dental plaque instead of saliva as substrate could explain the low *L. acidophilus* detection levels found during the study.

### 4.3. Infective Endocarditis (IE) and Oral Bacteria

Concerning the risk of IE in children with CHD, Topcuoglu et al. [42] examined saliva samples from children with CHD for the presence of *Streptococcus mutans* serotype *k*, a strain that was found in significantly greater amounts in heart valve specimens from patients with subacute IE [47]. There was no significant difference in the composition of salivary cariogenic microorganisms between children with CHD and healthy controls, but *Streptococcus mutans* serotype *k* was only found in CHD cases [42]. Due to the small sample sizes in both groups, further studies with larger numbers of cases are required to draw final conclusions about the prevalence of *Streptococcus mutans* serotype *k* in children with CHD.

Bozdogan et al. examined the presence of *Aggregatibacter actinomycetemcomitans*, a Gram-negative bacterium from the HACEK group, in samples of saliva and cardiac tissue in relation to the role of HACEK group bacteria as a cause of IE. There was no significant difference in the presence of this bacterium in saliva samples between children with CHD and healthy controls; however, children with CHD had a higher amount of colony-forming units despite having healthier gingiva [41].

If oral bacteria are suspected in heart tissue samples, it is reasonable to assume that these bacteria should also be detectable, at least temporarily, in the blood in the form of bacteremia. During the literature search for the present article, no studies could be found in which human infant blood was examined for oral bacteria.

At this point, however, it must be emphasized that a limitation of the results of the included studies on the topic of IE is that primarily young children with CHD were integrated into the studies, in whom the oral microbiome changes due to various external factors, such as the frequent use of antibiotics.

Steelman et al. published a study in 2000 containing evidence of a correlation between the severity of gingival inflammation and the presence of specific HACEK bacteria in both infants with CHD and healthy individuals [35].

Steelman et al. (2003) found no correlation between the number of HACEK microbes and the degree of gingival inflammation in children with CHD, whose gingival inflammation was comparable with that of healthy controls [36]. These findings may be explained by the fact that the HACEK group is part of the normal oral flora [29,30], highlighting the importance of using prophylactic antibiotics prior to invasive dental treatment in patients with a high risk of IE, even if there is no acute infection in the oral cavity.

### 4.4. Influence of Medications on the Oral Microflora

However, antibiotic resistance increases when they are used frequently [48]. Koh et al. (1986) focused [37] on amoxicillin-resistant *Streptococcus* strains in the dental plaque of infants with CHD and adults with a history of rheumatic fever. Children and adults in good health served as control groups. Comparing the two test groups with the control groups, the results revealed that the percentage of antibiotic-resistant *Streptococcus* strains was elevated in the two test groups [37].

The proportion of *Streptococcus* strains resistant to 1 mg/mL amoxicillin was more than twice as high in adults with a history of rheumatic fever compared with healthy controls. The authors assume that this is owing to the routine prophylactic administration of antibiotics to the test group [37].

In addition, Folwaczny et al. (2019) demonstrated that adults with CHD who require antibiotic prophylaxes prior to specific dental treatments have higher DMFT values and greater radiographic bone loss despite lower incidences of caries and periodontitis in comparison with healthy adults [49].

Torres et al. (2001) discovered a correlation between the frequent use of antibiotics and decreased *Lactobacillus* species counts, which occurred when antibiotics were regularly consumed. This correlation was not observed [43] for *Streptococcus mutans*.

These multiple findings lead to the conclusion that physicians should be aware of appropriate antibiotic use and should consider that in patients with CHD, a history of regular antibiotic use can potentially influence therapeutic outcomes and cause complications.

Diuretics, which can affect the rate of salivation [50], are also frequently used as an additional medication in pediatric patients with cardiomyopathies [51]. Rosén et al. (2010) did not find any significant differences in *Streptococcus mutans* or *Lactobacillus* spp. counts between children with CHD taking ACE inhibitors and/or diuretics and healthy children, but the total viable bacteria count was significantly higher in the healthy controls [39]. This may indicate a shift in the balance of bacterial diversity in children with CHD, which may be caused by a reduced saliva flow [52], especially considering that 29% of the CHD group in the study by Rosén et al. had very low salivary secretions, whereas the control infants did not [39].

### 4.5. Subgroup Factors

Several studies conducted subgroup analysis or examined additional groups in addition to children with CHD and healthy controls.

Steelman et al. (2000) and Steelman et al. (2003) distinguished cyanotic from acyanotic congenital heart disease infants. These subgroups did not differ significantly with regard to the presence of *Aggregatibacter actinomycetemcomitans* and *Eikenella corrodens* pathogens [35,36].

Ajami et al. (2015) also investigated a group of children with acquired cardiac diseases, in addition to healthy controls and children with CHD. There was no correlation between the type of heart disease and the presence of *Streptococcus mutans* in salivary samples, but children with acquired heart diseases had substantially *lower Streptococcus mutans* counts and lower DMFT scores [44].

Concerning the question of whether dental preventive treatment can compensate for oral dysbiosis in children with CHD, Suvarna et al. (2011) were able to demonstrate a reduction in CFUs of *Streptococcus mutans* as well as improved oral health after a preventive treatment consisting of local antibacterial agents, pits and fissure sealants, topical fluoride application, and oral hygiene education for children with CHD and their parents [11].

Considering that younger patients with increased dental treatment needs frequently receive treatments under general anesthesia, which is a risky approach in children with CHD [53] and also includes inpatient admission and an economic burden [54], it is all the more crucial that preventive treatments and oral health education be utilized to avoid these highly invasive procedures.

In summing up the findings of the present review, some evidence for differences in the oral bacterial spectrum of individuals with CHD, as well as a large number of potential influencing factors, were identified. However, only one of the included studies [40] examined a broad spectrum of bacteria as opposed to focusing on a few specific bacteria.

## 5. Conclusions

There is some evidence for alterations in the oral microflora in children with CHD due to physiopathological and treatment-related factors, but additional research is required to validate these findings. The changes in oral flora as an indicator of a general infection could be helpful for the prevention of endocarditis, especially for this group of vulnerable patients, and thus also increase the quality of life of these patients. In addition, future research should investigate the influencing factors that may be present in children with CHD.

## Figures and Tables

**Table 1:** Items of the research question according to the PICO scheme (CHD = congenital heart disease).

Population	Children (0–18 Years of Age) with CHD.
Intervention	Gaining of samples of the oral cavity and investigation of these samples for bacteria.
Comparison	Healthy children (0–18 years of age) without CHD.
Outcome	Information on the composition of the bacterial colonization of the oral cavity.

**Table 2:** Characteristics of included studies (CHD = congenital heart disease).

	Saliva								
Study	Place of Study (Country)	Methods	Test Group	Control Group	Medium	Sampling	Examined Bacteria	Results	Significance
Pourmoghaddas et al. (2018) [45]	Iran	Culture, determination of number of CFUs	68 children with CHD (mean age = 7.4 years)	74 healthy children (matched by age and sex; [mean age = 7.8 years])	Saliva	2 mL was taken	*Streptococcus mutans*, *Lactobacillus* species	Significantly higher number of CFUs of *Streptococcus mutans* in saliva samples of the healthy control group. No significant difference in the amount of CFUs of *Lactobacillus* spp. No significant correlation between dmf-t values and the number of CFUs of *Streptococcus mutans and Lactobacillus* spp. No significant correlation between *Streptococcus mutans/* *Lactobacillus* spp. and gingival bleeding index.	*p* = 0.03 for mean saliva colony counts of *Streptococcus mutans*. *p* = 0.3 for the mean colony count of *Lactobacillus* spp.
Bozdogan et al. (2016) [41]	Turkey	PCR, agarose gel electrophoresis	25 children with CHD undergoing elective surgery (3–12 years of age)	25 healthy children (age and gender-matched)	Stimulated saliva	Children chew a sugar-free gum (paraffin) and spit into a sterile container. Saliva collection was carried out for 5 min. Smaller children (unable to chew): Swab samples from dental surfaces (sterile swab sticks).	*Aggregatibacter Actinomycetemcomitans* and its JP2 clonal strains	No significant differences between groups with respect to the presence of *Aggregatibacter Actinomycetemcomitans* in saliva samples. Only one positive result for a JP2 clonal strain in 1 saliva sample of the control group.	*p* > 0.05 for *Aggregatibacter Actinomycetemcomitans* in saliva samples
Ajami et al. (2015) [44]	Iran	Culture, determination of number of CFUs	50 children with CHD (+16 children with acquired heart disease), 3–12 years of age	50 healthy children, 3–12 years of age	Saliva	15 s sampling via a swab from the lingual area of the mandibular teeth	*Streptococcus mutans*	Significantly higher number of CFUs of *Streptococcus mutans* in children with CHD.	*p* = 0.026
Topcuoglu et al. (2013) [42]	Turkey	Culture, PCR (species-specific 16S rRNA gene sequences, serotype-specific *rgpF* gene sequences)	25 children with CHD undergoing elective surgery for congenital heart defects with cardiopulmonary bypass (3–12 years of age)	25 healthy children (age and gender-matched)	Stimulated saliva	Saliva sampling analogous to [6]	Culture: *Streptococcus mutans* and *Lactobacillus* spp. PCR techniques: *Streptococcus mutans* (including determination of serotype *k*, for test group only)	No significant differences between the study and control groups with respect to the numbers of investigated microorganisms and the detection of *Streptococcus mutans* in saliva samples. Determination rate of *Streptococcus mutans* serotype *k* was 12% (test group).	*Streptococcus mutans*: *p* = 0.269 *Lactobacillus* spp.: *p* = 0.517
Hansson et al. (2012) [38]	Sweden	Culture, determination of number of CFUs	11 children with severe CHD (6, 9, and 12 months of age)	22 formula-fed healthy children (same age and gender)	Saliva	At 6, 9, 12 months of age Wooden, saline-wetted cotton swab: streaked along buccal mucosa in the anterior part of the mandible and the maxilla	*Streptococcus mutans*, *Lactobacillus* spp.	Significantly higher number of CFUs of *Streptococcus mutans* and significantly higher *Streptococcus mutans* ratio of total viability count in the test group at the age of 12 months (but not at 6 and 9 months). At 12 months of age, 90% of the test group and 54% of the control group showed detectable levels of *Streptococcus mutans*. No sample in the test group showed detectable levels of *Lactobacillus* spp. (3 samples og the whole period in the control group showed detectable levels).	Rate of *Streptococcus mutans* colonisation: 6 months of age: *p* > 0.05 9 months of age: *p* > 0.05 12 months of age: *p* < 0.01
Suvarna et al. (2011) [11]	India	Culture, determination of number of CFUs	74 children with CHD (5–16 years of age)	30 healthy siblings (same age group)	Unstimulated saliva	Collection of 1 mL of whole unstimulated saliva in sterile tubes	*Streptococcus mutans*	Higher mean values for the amount of CFUs of *Streptococcus mutans* in the test group.	Significance level is not reported.
Rosén et al. (2010) [39]	Sweden	Culture, determination of number of CFUs	24 children with CHD, medicated with ACE inhibitors and or diuretics (6–19 years of age)	1 friend of the same age and gender as each participant in the test group	Stimulated saliva	5 min of chewing paraffin wax and collecting all saliva produced in a test tube	*Streptococcus mutans*, *Lactobacillus* spp., Total viable count of bacteria	Significantly higher total viable count of bacteria in the test group.For levels of *Lactobacillus* spp. and *Streptococcus mutans*, and *Streptococcus mutans* ratio of the total viable count of bacteria, no significant differences could be found.	Total viable count of bacteria: *p* < 0.05 Levels of *Lactobacillus* spp. and *Streptococcus mutans*: *p* > 0.05
Torres et al. (2001) [43]	Brazil	Culture, determination of number of CFUs	20 cardiac children (15 of them with CHD; 6–14 years of age)	15 healthy children (6–14 years of age)	Stimulated saliva	Collection after 10 min of paraffin chewing stimulation: spitting into a universal tube	*Streptococcus mutans*, *Lactobacillus* spp.	No significant differences between groups with respect to the number of CFUs of *Streptococcus mutans*. Saliva of cardiac children showed a significantly lower number of CFUs of *Lactobacillus* spp. than samples from the control group (in relation to the use of antibiotics).	*Streptococcus mutans*: *p* > 0.05 *Lactobacillus* spp.: *p* < 0.05
Franco (1996) [6]	United Kingdom	Culture, determination of number of CFUs	60 Children with CHD (2–16 years of age)	60 healthy siblings and other healthy children (matched by age, gender, ethnicity, social class)	Unstimulated saliva	Sampling of 3 mL of unstimulated saliva by gently spitting into a universal tube. Very young children/children unable to spit: collection of saliva via a syringe without a needle.	*Streptococcus mutans*, *Lactobacillus* spp.	No significant differences between groups of children. In general, the number of CFUs of salivary *Streptococcus mutans* was higher than plaque counts. A large proportion of children had no detectable *Lactobacillus* spp. in saliva (40.6%) and plaque (68.8%).	No significant differences, but the significance level is not reported.
	**Subgingival samples**								
**Study**	**Place of study (country)**	**Methods**	**Test group**	**Control group**	**Medium**	**Sampling**	**Examined bacteria**	**Results**	**Significance**
Steelman et al. (2003) [36]	United States of America	Culture, DNA probe tests	12 children with CHD (2,5–10 years of age)	12 healthy children (age and gingival index matched; 2–13 years of age)	Subgingival samples	Insertion of a sterile endodontic paper point into the gingival sulcus	*Hemophilus* spp., *Actinobacillus* spp., *Cardiobacter* spp., *Eikenella* spp. and *Kingella* spp. (HACEK) microbes	9 of 12 children from the test group and 3 of 12 children from the control group showed *Eikenella corrodens* microbes. 3 children from the test group and 0 children from the control group showed *Aggregatibacter actinomycetemcomitans* microbes.	*Eikenella corrodens* microbes: *p* < 0.05 Significance for other bacterial species is not reported.
Steelman et al. (2000) [35]	United States of America	Culture, determination of number of CFUs	12 children with CHD (1.5–8 years of age)	12 healthy children (age and sex-matched; 2–8 years of age)	Subgingival samples	Insertion of a sterile paper point into the gingival sulcus in two separate areas with the greatest degree of gingival inflammation	Total *Streptococcus* spp. and the HACEK group	Statistically significantly higher value of *Actinobacillus actinomycetemcomitans* and higher (but not statistically significantly higher) values of *Eikenella* spp. in samples from the test group. Levels of *Actinobacillus actinomycetemcomitans* and *Eikenella* spp. increased with a higher degree of inflammation of the gingiva (gingival index). Even at a lower gingival index, the absolute number of CFUs was greater in samples in the test group. No significant difference in total *Streptococcus* spp. counting between groups.	*Actinobacillus actinomycetemcomitans*: *p* < 0.05 *Eikenella corrodens* microbes: *p* = 0.06 Total *Streptococcus* spp: *p* < 0.05
	**Supragingival dental plaque (biofilm)**								
**Study**	**Place of study (country)**	**Methods**	**Test group**	**Control group**	**Medium**	**Sampling**	**Examined bacteria**	**Results**	**Significance**
Mohamed Ali et al. (2017) [22]	Sudan	DNA extraction and purification, bacterial DNA detection (RT-qPCR), bacterial species detection (checkerboard DNA-DNA hybridization)	80 children with CHD (3–12 years of age)	80 healthy children (matched to children in the test group by age, sex, and the use of antibiotics; 3–12 years of age).	Dental plaque (biofilm)	Paper points were scraped against the tooth (under the isolation of saliva)	Detection and quantification of *Streptococcus mutans*, *Streptococcus sanguinis*, and *Lactobacillus acidophilus*, detection of 40 additional bacterial species	*Streptococcus mutans*: 87.5% of CHD cases versus 88.7% of controls. *Streptococcus sanguinis*: 97.5% for both groups. *Lactobacillus acidophilus*: only 1 case per group. 18 bacterial species were more frequently detected, significantly higher mean number of CFUs in the test group (including *Porphyromonas gingivalis* of the red complex and seven species of the orange complex). Correlation between the amount of CFUs of bacterial species and gingivitis for *Tanerella forsythia* (red complex), *Camphylobacter rectus,* and *Fusobacterium nucleatum* (subspecies *polymorphum*, *vincentii*, *nucleatum*) from the orange complex only among cases in the test group. Correlation between the number of CFU bacterial species and gingivitis for *Centruroides gracilis*, *Prevotella intermedia*, *Prevotella nigrescens*, and *Selenomonas noxia* (all of the orange complex) only among cases of the control group.	*Streptococcus mutans*: *p* = 0.019 *Streptococcus sanguinis*: *p* = 0.227
Franco (1996) [6]	United Kingdom	Culture, determination of number of CFUs	60 Children with CHD (2–16 years of age)	60 healthy siblings and other healthy children (matched by age, gender, ethnicity, social class)	Dental plaque (biofilm)	Gently flossing between the molar teeth in the primary and/or secondary dentition in all four quadrants (until a visible amount of plaque was obtained). If not possible: obtaining plaque with a small ball-ended probe from as close to the approximal region as possible	*Streptococcus mutans*, *Lactobacillus* spp.	No significant differences between groups of children. In general, the number of CFUs of salivary *Streptococcus mutans* was higher than plaque counts. A large proportion of children had no detectable *Lactobacillus* spp. in saliva (40.6%) and plaque (68.8%).	*Streptococcus mutans*, *Lactobacillus* spp.: No significant differences, but the significance level is not reported.
Koh et al. (1986) [37]	United Kingdom	Culture, determination of CFUs, Gram-staining, Identification of bacteria on the basis of morphology	20 children with CHD (10 female, 10 male; 3–18 years of age; mean age = 10.0 years)	20 healthy children (12 female, 8 male; 5–15 years of age; mean age = 11.4 years)	Supragingival dental plaque	Using miniature sterile swabs of calcium alginate bacteriological wool to collect plaque from the supragingival margin of a maximum of tooth surfaces (rotation)	Amoxicillin-resistant oral *Streptococcus* spp.	High percentage of children from the test group showed amoxicillin-resistant oral *Streptococcus* spp. 0 subjects from the control group and 1 subject from the test group showed *Streptococcus* spp. resistant to 10 mg/liter amoxicillin. With respect to *Streptococcus* spp. resistant to 1 mg/liter amoxicillin: no significant differences between groups. 80% of children with CHD and 35% of the control group showed *Streptococcus* spp. To be resistant to 1 mg/L amoxicillin.	*Streptococcus* spp. resistant to 1 mg/L amoxicillin: No significant differences, but significance level is not reported.
	**Carious dentinal material**								
**Study**	**Place of study (country)**	**Methods**	**Test group**	**Control group**	**Medium**	**Sampling**	**Examined bacteria**	**Results**	**Significance**
Schulz-Weidner et al. 2021 [40]	Germany	DNA Extraction and 16S RNA Gene Amplicon Sequencing (V4)	11 preschool children with CHD and with Early Childhood Caries (2–6 years of age)	9 preschool children (maximum ASA class I) with Early Childhood Caries (almost balanced by gender and carious status; 2–6 years of age)	Carious dentinal material	Collection by a sterile excavator	Whole bacterial microbiome	3 distinct clusters for all samples; one of them only containing samples from the test group. Significantly higher abundance of *Fusobacterium* spp., *Prevotella* spp., *Capnocytophaga* spp. and *Oribacterium* spp. OTUs in samples from the test group. *Lactobacillus* spp. and *Rothia* spp. as discriminative features for the control group.	*Fusobacterium* spp., *Prevotella* spp., *Capnocytophaga* spp., *Oribacterium* spp., *Lactobacillus* spp. and *Rothia* spp.: *p* < 0.05
	**Cardiac tissue samples**								
**Study**	**Place of study (country)**	**Methods**	**Test group**	**Control group**	**Medium**	**Sampling**	**Examined bacteria**	**Results**	**Significance**
Bozdogan et al. (2016) [41]	Turkey	PCR, agarose gel electrophoresis	25 children with CHD undergoing elective surgery (3–12 years of age)	No control group	Cardiac tissue	Collected under aseptic conditions during cardiac surgery	*Aggregatibacter Actinomycetemcomitans* and its JP2 clonal strains	No bacterial DNA was found in cardiac tissue samples in the test group.	/
